# Repeatability and reproducibility of retinal nerve fibre layer thickness measurements with the iVue-100 optical coherence tomographer

**DOI:** 10.4314/ahs.v18i2.15

**Published:** 2018-06

**Authors:** Nishanee Rampersad, Rekha Hansraj

**Affiliations:** Discipline of Optometry School of Health Sciences, College of Health Sciences, University of KwaZulu-Natal, Durban, South Africa

**Keywords:** Retinal nerve fibre layer thickness, optical coherence tomography, repeatability, reproducibility, iVue-100 OCT

## Abstract

**Background:**

Accurate and repeatable measurements of the retinal nerve fibre layer (RNFL) thickness are important in the diagnosis and management of glaucoma and other disorders.

**Objective:**

To assess the repeatability and reproducibility of the iVue-100 optical coherence tomographer (OCT).

**Methods:**

The thickness of the RNFL was measured for 50 healthy participants using the iVue-100 OCT. Although both eyes per participant were measured, only right eyes were analysed here. Repeatability and reproducibility of the iVue-100 OCT were assessed using the intraclass correlation coefficient (ICC), coefficient of variation (CoV), paired t-tests and Bland-Altman analysis.

**Results:**

Good intra-observer repeatability was obtained as indicated by the ICC of observer 1 (range: 0.941 – 0.976) and observer 2 (range: 0.829 – 0.953) as well by the CoV of observer 1 (range: 0.098 – 0.137) and observer 2 (0.091 – 0.132). In terms of inter-observer reproducibility, significant differences (p< 0.05) in mean measurements between the observers were noted for the average RNFL readings and in the superior and inferior quadrants as assessed with paired t-tests. Even though significant inter-session differences were found for the average RNFL thickness and the superior quadrant (p = 0.003 and p = 0.013, respectively), excellent ICCs were obtained for inter-session reproducibility (range: 0.914 – 0.979).

**Conclusion:**

The iVue-100 OCT demonstrated good repeatability and reproducibility for RNFL thickness measurements.

## Introduction

The retinal nerve fibre layer (RNFL) lies between the ganglion cell layer and inner limiting membrane of the retina.^1^ As it comprises of the axons of the ganglion cells, it is sometimes referred to as the axon layer. These axons transmit the chemical message from the retina to synapse at the lateral geniculate nucleus before reaching the occipital cortex. There is a varied but definite retinotopic arrangement of the nerve fibres as they (neural impulses) make their way through the visual pathway in preparation for impulses to be received optimally at the occipital cortex. The thickness of the RNFL on the optic nerve head is found to vary in the four quadrants (superior, inferior, nasal and temporal) being thickest in the inferior quadrant.^1^,^2^

The thickness of the RNFL varies with age, ethnicity, axial length and optic disc area apparently but not with gender.^3^ Various ocular pathologies including retinitis pigmentosa and glaucoma result in an altered RNFL thickness^4^–^6^ making the clinical measurement of RNFL thickness of importance in both the diagnosis and management of ocular conditions.

Glaucoma is the second leading cause of blindness accounting for 10.1% of global blindness.^7^,^8^ In Africa, primary open-angle glaucoma is the predominant form of glaucoma and is likely to affect 8.0 million African individuals by the year 2020.^9^ Glaucoma is associated with thinning of the retinal nerve fibre layer (RNFL) and loss of ganglion cells.^10^,^11^ Consequently, assessment of the RNFL is important in the screening, diagnosis and monitoring of glaucoma since RNFL changes preceed visual field changes.^12^,^13^ Systemic conditions such as multiple sclerosis have also been associated with RNFL thinning.^14^

Common methods used to assess the RNFL include scanning laser polarimetry, confocal scanning laser ophthalmoscopy, fundus photography and optical coherence tomography.^10^ Optical coherence tomography, a non-invasive method that produces high-resolution, cross-sectional micrometre-scale images of biological structures, is frequently used to assess the RNFL.^10^,^15^ The principle of OCT is analogous to ultrasound except that reflected light waves and not reflected sound waves are measured and analysed.^16^ Compared to traditional time-domain OCT devices that were introduced in the early 1990s, the newer generation Fourier/spectral-domain OCT devices provide higher axial resolutions, faster scanning speeds and better eye movement tracking.^17^,^18^

Repeatability describes the ability of an instrument to provide consistent measurements for a single observer under the same conditions on the same visit. In addition, reproducibility of refers to consistent measurements when an instrument is used by two different observers (inter-observer repeatability) and in two separate sessions (inter-session repeatability).

Good repeatability and reproducibility are characteristics of an instrument for it to be useful in research and in clinical practice.^19^ Previous studies^19^–^22^ on the repeatability of RFNL thickness using OCT included time-domain and Fourier domain OCTs and their comparisons. However, many of these studies were on American and European samples with little to no inclusion of Asian and African individuals.^3^ Furthermore, to the researchers' best knowledge, limited studies have investigated the repeatability of RNFL measurements using the iVue-100 OCT. Therefore, this study investigated repeatability of the iVue-100 OCT for RNFL thickness measurements in a South African sample.

## Methods

Convenience sampling was used to recruit 50 participants, of all races, gender and ages in this observational cross-sectional study. The study followed the tenets of the Declaration of Helsinki and all participants were required to give written informed consent. Ethical approval was obtained from the Biomedical Research and Ethics committee (BE024/13) of the UKZN. All participants had normal corneal topography, visual acuity of at least 20/20 (aided or unaided) and no history of ocular injury and/or surgery.

**Figure 1:**
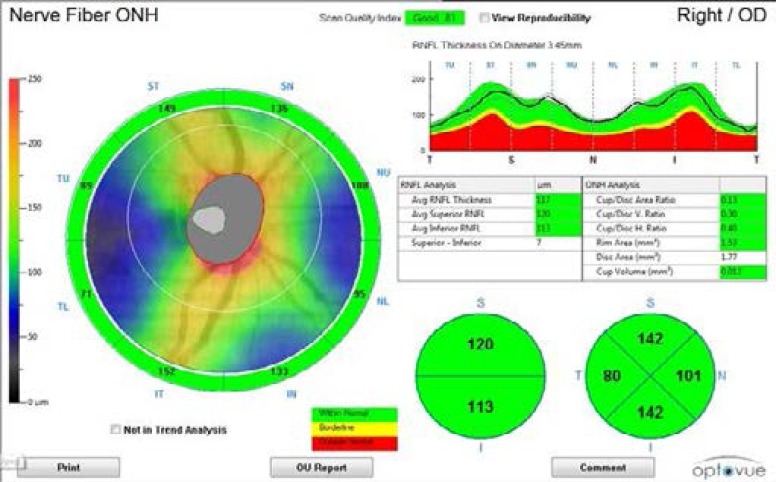
RNFL thickness display of the optic nerve head scan

The RNFL thickness was scanned and measured using the Optovue iVue-100 OCT device on both eyes per participant most frequently before midday, and over a period of 10 weeks. This Fourier-domain OCT device operates at a scanning speed of 26000 A-scans per second with a frame rate of 256 to 4096 A-scans per frame.^23^ The axial and transverse resolutions of the iVue-100 OCT device are 5 µm and 15 µm respectively (manual). A pre-programmed algorithm in the iVue-100 OCT device automatically determines the RNFL thickness as the distance in micrometres between the anterior and posterior boundaries of the highly reflective retinal layer.^10^,^23^ The optic nerve head scan protocol which consists of 12 radial (3.4 mm) line scans of 459 A-scans each was used to measure the RNFL thickness. In this scan protocol, the RNFL thickness is measured along a 3.45 mm diameter circular ring centred on the optic disc.^23^ The optic nerve head scan automatically determines the average RNFL thickness in the superior (46° to 135°), nasal (136° to 225°), inferior (226° to 315°) and temporal (316° to 45°) quadrants as well as the global RNFL thickness displayed as the “average RNFL thickness” on the scan (Figure 1).

A laptop screen was used to monitor the real-time image of the participant's eye and RNFL when scanning while the participant fixated an internal fixation target. The participant's head was stabilized using forehead and chin rests. Repeat scans were taken if the scan quality index was less than 27 and/or labelled as ‘poor’ on the laptop screen display.^23^ For the assessment of intra-observer repeatability, the optic nerve head scanning protocol was repeated three times on the same participant. Inter-observer repeatability was assessed by having a second observer repeat the optic nerve head scanning protocol on the same participant at each visit. For inter-session repeatability, observer one repeated the scanning protocol on the same participant on another day. Each observer re-aligned the iVue-100 OCT device before capturing each scan.

Data were analysed using the Statistical Package for Social Sciences (SPSS) version^24^. Repeatability of the measurements was reported in terms of the intraclass correlation coefficient (ICC) and coefficient of variation (CoV). Bland-Altman analysis and t-tests were also used to report on the inter-observer and inter-session repeatability. The significance level was set at 95% with p-value of ≤ 0.05.

## Results

### Demographics

The sample consisted of 50 participants with almost twice as many females (n= 32) than males (n = 18). The ages of the participants ranged between 18 and 51 years with a mean of 23.88 ± 6.93 years (median age = 22.00 years). The majority of the participants were Indian (54%) followed by Black (36%), White (8%) and Asian (2%). Half of the participants has spherical equivalent refractive errors less than or equal to 0.50 D. The spherical equivalent of the right eye ranged from −8.38 to +1.63 D and that of the left eye ranged from −9.63 to +3.00 D. Due to the limited sample size the correlation between refractive error and nerve fibre layer was not considered in this study. The right and left eye measurements correlated in the four quadrants (0.940 ≥ r ≥ 0.750) with differences ≤ 4.60, therefore only the right eye measurements were analysed.

### Intra-observer repeatability

Table 1 shows the ICC together with the 95% confidence intervals and the CoV for each observer for RNFL quadrants.

**Table 1:** Intraclass correlation coefficient with 95% confidence intervals and CoV for each observer, for RNFL thickness measured for the different quadrants of the right eye

	OBSERVER 1		OBSERVER 2	
	ICC (95% CI)	CoV	ICC (95% CI)	CoV
**Average**	0.976 (0.962–0.986)	0.098	0.953 (0.925–0.972)	0.091
**Superior**	0.968 (0.948–0.981)	0.137	0.919 (0.870–0.952)	0.125
**Inferior**	0.972 (0.956–0.983)	0.111	0.872 (0.795–0.923)	0.108
**Nasal**	0.941 (0.906–0.965)	0.134	0.829 (0.727–0.898)	0.140
**Temporal**	0.958 (0.932–0.975)	0.132	0.918 (0.868–0.951)	0.132

Excellent intra-observer repeatability was noted as the ICCs for observer one ranged from 0.941 to 0.976 and that for observer two from 0.829 to 0.953. The highest ICCs were noted for the average RNFL thickness and the lowest(0.941 and 0.829) were noted in the nasal quadrant for the two observers. The CoV for observer one ranged from 0.098 to 0.137 and that of observer two, from 0.091 to 0.132 indicating good intra-observer repeatability.

### Inter-observer repeatability

For the different quadrants of the right eyes. Table 2 also shows the Bland and Altman limits of agreement (LoA). The Paired t-tests were used to determine the t-values and p-values as an indication of significant differences between observers.

**Table 2:** The mean differences, and their standard deviations, of RNFL thickness (µm) between two observers, Bland and Altman upper and lower LoA, *t*-values and *p*-values from the Paired *t*-tests and the ICC

	Mean diff (µm)	SD(µm)	Upper LoA	Lower LoA	*t*-value	*p*-value	ICC
**Average**	1.047	2.959	6.847	−4.753	2.501	0.016	0.976
**Superior**	2.667	6.998	16.383	−11.049	2.640	0.011	0.954
**Inferior**	2.507	8.121	18.424	−13.410	2.183	0.034	0.912
**Nasal**	−0.253	6.631	12.744	−13.250	−0.270	0.788	0.906
**Temporal**	−0.403	4.993	9.383	−10.189	−0.559	0.579	0.932

There were no statistically significant differences in means between the two observers for the nasal and temporal quadrants (p > 0.05). While statistically significant differences in means were found for the superior, inferior and average RNFL (p<0.05), the mean differences were less than 3 µm (Table 2). The ICCs ranged between 0.906 and 0.976 indicating excellent repeatability.

**Figure 2:**
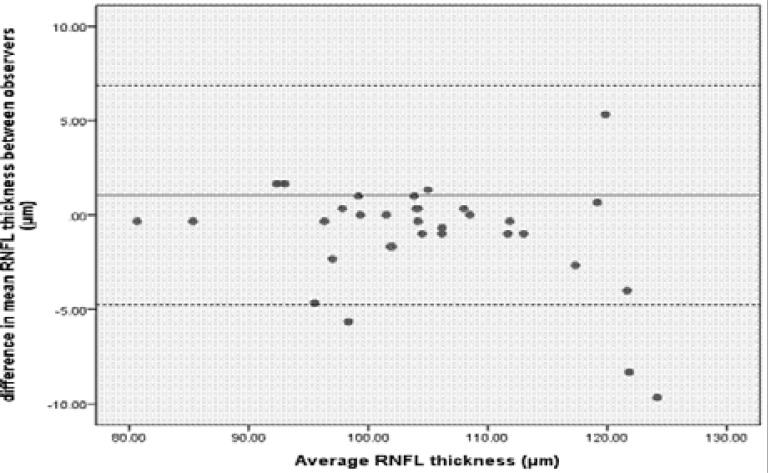
Bland-Altman plot comparing RNFL thickness measurements of the right eyes taken by observer one and observer two. The solid line represents the mean difference and the dashed lines represent the upper and lower LoA. Three points were outside the 95% LoA region.

Bland-Altman plots were used to graphically compare the average RNFL thickness measurements of the two observers (Figure 2). All measurements were within the 95% LoA with the exception of three. The mean difference was small at 1.047µm.

### Inter-session repeatability

The measurements of observer one were used to determine the inter-session repeatability as this observer had completed more inter-session repeat readings which were taken on two separate sessions. The interval between sessions 1 and 2 ranged between 1 and 70 days. The mean difference of the RNFL readings ranged from 0.222 µm to 4.061 µm (Table 3) with the ICC ranging between 0.914 and 0.979. Paired t-tests were used to determine the t-values and p-values as an indication of significant differences between sessions. Statistically significant differences in the mean RNFL thickness measurements between sessions were noted in the average and superior quadrant.

**Table 3:** The mean differences and their standard deviations, of RNFL thickness (µm) for observer one taken over two sessions, Bland and Altman upper and lower LoA, and *t*-values and *p*-values from Paired *t*-tests and ICC

	Mean diff (µm)	SD(µm)	Upper LoA	Lower LoA	*t*-value	*p*-value	ICC
**Average**	1.465	2.625	6.610	−3.680	3.206	0.003	0.979
**Superior**	4.061	8.841	21.389	−13.267	2.639	0.013	0.914
**Inferior**	1.455	5.321	11.884	−8.974	1.570	0.126	0.969
**Nasal**	0.222	4.421	8.887	−8.443	.289	0.775	0.942
**Temporal**	0.162	3.654	7.324	−7.000	.254	0.801	0.965

**Figure 3:**
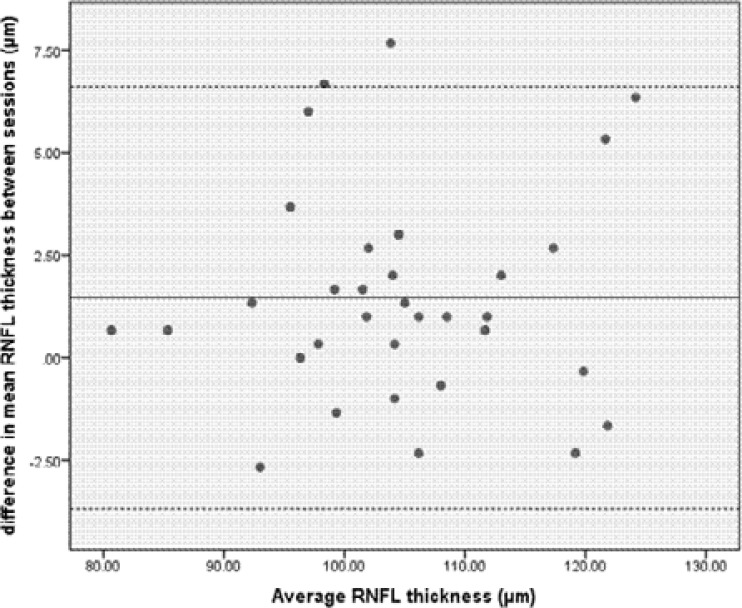
Bland-Altman plot comparing average RNFL measurements of the right eyes taken by observer one in two separate sessions. The solid line represents the mean difference and the dashed lines represent the upper and lower LoA.

Bland-Altman plots were used to graphically compare the average RNFL thickness measurements taken by observer one over two sessions (Figure 3). All measurements were within the 95% LoA with the exception of only one measurement. The mean difference was less than 1.465 µm.

## Discussion

The estimation of an instrument's repeatability and reproducibility is of paramount importance^19^ particularly one that is being used for clinical and research applications. Accurate and reliable measurements of the RNFL thickness is necessary in both the diagnosis and management of retinal disorders particularly glaucoma and retinitis pigmentosa.^4^–^6^ Optical coherence tomography provides an accurate, non-invasive and rapid quantitative assessment of RNFL thickness.^24^ The iVue-100, a spectral domain OCT, has gained popularity in its usage in the clinical field, however it's repeatability and reproducibility for measurement of RNFL thickness has not been extensively studied previously.

Repeatability of the iVue-100 OCT was assessed using the ICC and CoV as the indicators. The ICC can lie anywhere between 0 and 1, with a higher ICC indicating minimal fluctuation in repeated readings.^5^,^19^ Furthermore, an ICC of between 0.81 and 0.99 represents good agreement between repeated measurements.^25^ The ICCs of both observers in this study were greater than 0.8 thereby implying good intra-observer repeatability.

The CoV indicates the measurement variability in relation to the mean and has been used in other repeatability studies.^5^,^19^ A CoV <0.1 indicates high repeatability.^20^ The CoVs for both observers ranged from 0.091 to 0.137 again indicating good intra-observer repeatability. In the present study, the best intra-observer repeatability was noted for the average RNFL thickness measurements for both observer 1 and 2 in terms of the ICC and CoV which is consistent with the findings of other studies.^19^–^22^ The poorest intra-observer ICC was noted for the superior quadrant for observer 1 and the nasal quadrant for observer 2. Similar discrepancies have been reported for intra-observer repeatability as González-García et al^19^ and Garcia-Martin et al^21^ found the lowest intra-observer ICC for RNFL thickness measurements in the nasal quadrant while Paunescu et al^22^ found the inferior quadrant to have the lowest ICC. It should be noted that the current study calculated the ICC and CoV based on the mean difference between the measurements, however previous studies have used the actual mean RNFL thickness.

Clinically when monitoring the progression of a disorder it is essential that the smallest changes are detected which depend on the instrument reproducibility.^5^,^19^ The Paired t-tests and Bland-Altman analysis were used in addition to the ICC to assess the reproducibility, which has been the case in previous studies.^20^,^22^,^25^

The ICC indicated excellent inter-observer and inter-session repeatability (all ICCs ≥ 0.906). However, the Paired t-tests noted significant inter-observer mean differences for the average RNFL thickness (1.047 µm), superior (2.667 µm) and inferior (2.507 µm) quadrants. Assuming the normal average RNFL thickness as being approximately 104.27 µm and that an 8.4% (8.75 µm) RNFL loss is required before visual field changes are detected^26^ these mean differences of less than 3 µm as found in the present study are possibly not clinically significant. Similar mean differences for inter-observer repeatability were reported by Gürses-Özden et al^18^ of 2.9 µm for the superior quadrant and 3.3 µm for the inferior quadrant using the fast scan protocol on the Carl Zeiss Meditec OCT-3, however, their Paired t-tests did not show these differences to be significant. Also, the sample size for the study by Gürses-Özden et al^20^ was only 10 subjects and the pupils were dilated unlike in the present study.

In our study, significant inter-session differences as indicated by Paired t-tests were only noted for the average RNFL thickness (1.465 µm, p = 0.003) and in at superior quadrant (4.061 µm, p = 0.013). Even though Gürses-Özden et al^19^ found statistically insignificant intersession RNFL thickness measurements, the mean intersession differences were higher (range: 1.067 µm to 6.800 µm) than that found in our study. Garcia-Martin et al^21^ reported much smaller inter-session mean differences (range: 0.2 µm to 1.27 µm) using a Fourier-domain OCT as in the present study unlike Gürses-Özden et al^20^ who used a time-domain device.

Bland-Altman analysis was further used to quantify the agreement between inter-observer and inter-session measurements for the average RNFL thickness. Generally agreement between two measurements is concluded if 95% of the data points lie within two standard deviations of the mean difference (LoA), when using this analysis.^27^,^28^ The LoA describe the variability of repeated measurements either between two observers and/or two different sessions. In the current study, good repeatability was obtained for inter-session repeatability as 97% of the data points were within the upper and lower LoA, but this was not the case for inter-observer repeatability as only 91% of the data points were within the LoA for the average RNFL thickness. The size of the LoA for inter-observer and inter-session repeatability were 11.60 and 10.29 µm, respectively. However, the Bland-Altman LoA should be interpreted not only statistically but also for their clinical significance.^29^,^30^ The mean differences for both inter-observer and inter-session repeatability were less than 2 µm and again may be clinically insignificant.

Previous OCT studies^20^,^31^–^33^ have noted the average RNFL thickness measurements to be the most reproducible and the least reproducible to be the nasal region which they related to low reflectance of light reaching the aperture in the nasal region and the greater number of blood vessels in the nasal peripapillary region compared to other peripapillary regions. In the current study, the average RNFL thickness was most reproducible in all instances, however while the nasal quadrant was the least reproducible for intra- and inter-observer repeatability, it was the superior quadrant that showed the least inter-session reproducibility. In spite of this, for the least reproducible quadrants the ICC were all greater than 0.905 and the mean differences less than 4.5 µm. The results of our study therefore suggest that while the iVue-100 OCT has good repeatability and reproducibility, variations in consistency of RNFL thickness measurements in all quadrants may occur.

Good repeatability and reproducibility of the iVue-100 OCT were demonstrated in 50 normal eyes, however future studies should focus on the repeatability of this instrument in larger samples and also in eyes particularly with glaucoma and possibly other conditions such as retinitis pigmentosa. Comparisons with other spectral-domain OCTs that may have different scanning speeds, axial resolution, signal strengths and algorithms would also be useful.
